# The Mediating Role of Psychological Capital between Motivational Orientations and Their Organizational Consequences

**DOI:** 10.3390/ijerph17134864

**Published:** 2020-07-06

**Authors:** Francisco Rodríguez-Cifuentes, Adrián Segura-Camacho, Cristina García-Ael, Gabriela Topa

**Affiliations:** 1 Department of Psychology, Rey Juan Carlos I University, 28933 Madrid, Spain; francisco.rcifuentes@urjc.es; 2 Department of Social, Developmental and Educational Psychology, University of Huelva, 21071 Huelva, Spain; adrian.segura@dpee.uhu.es; 3 Department of Social and Organizational Psychology, National Distance Education University (UNED), 28045 Madrid, Spain; cgarciaael@psi.uned.es

**Keywords:** motivational traits, Motivational Traits Questionnaire, psychological capital, organizational citizenship behaviors, counterproductive work behavior

## Abstract

Just as we can speak of different personality traits, it is also possible to identify distinct motivational traits, which may be related to a series of organizational consequences. In this sense, understanding how these traits are related to workers performance is fundamental. Specifically, the purpose of this study is to test the mediating role of psychological capital in the relationship between such traits and organizational citizenship behaviors and counterproductive work behaviors, which is expected to be more significant in the first case. The study was carried out using a panel design, with a sample group of Spanish employees aged over 40 (*n* = 741), in two waves (with a 4-month interval). The results support the hypothesis that psychological capital resources may play a mediating role in some of the relationships explored and that approach orientation traits are mainly related to a better performance, fostering organizational citizenship behaviors and diminishing counterproductive work behavior. The findings show that employees who develop their personal resources may have a positive impact on their organizations. The implications of this study for counseling practices are discussed.

## 1. Introduction

On what does employee performance depend? Many academics and organizations have sought to answer this question, not merely with a view to identifying causes, but also in order to achieve a real impact and thus improve performance. There are a number of primary reasons for this interest: (a) average workers’ age is increasing [1] and many of them remain in the workforce beyond retirement age [2], while capacities and motivations vary throughout a person’s life [3,4]; (b) performance is not only important at an organizational level, it also influences people’s self-concept and self-esteem [5]; (c) performance may be related to a desire to keep working for longer [6]; and (d) not everyone performs equally under the same conditions or for the same reasons [7]. It is therefore important to know “what lies behind performance” in order to encourage it.

Work motivation is understood here as one of the basic pillars for studying performance. However, attitude is not everything, and a series of skills are required not only to effectively carry out the tasks involved in the job itself but also to establish a working climate conducive to organizational success. The present study is therefore based on the idea that people differ on motivational traits, as Kanfer et al. [8] argue. At the same time, it is also based on Luthans et al.’s [9] development of the concept of psychological capital as a set of personal resources that can predict a series of results, including performance.

The aim of this paper is to analyze the potential mediating role of psychological capital in the relationship between motivational traits and performance in the form of extra-role behaviors (both positive and negative for the company).

The results of the study will enable us to establish whether the reasons for certain organizational behaviors can be found in stable aspects of the subjects’ personalities and whether these aspects are related to personal resources, which are, by definition, developable. Ultimately, the aim is to enable interventions similar to those currently being used to improve performance, but better adapted to the specific circumstances of each worker.

### 1.1. Motivational Orientations and Performance

Organizational performance is a subject of interest for a number of different disciplines. One branch of organizational psychology has focused on the role played by motivation in obtaining results. In their review, Kanfer and Chen [10] note that motivation has been studied from different perspectives, ranging from the initial approaches, based on the concept of necessity, to a more recent view of motivation as a process of resource allocation. One of the most widely accepted categorizations of goal orientation distinguishes between motivation oriented towards approach (e.g., achieving success) and motivation oriented towards avoidance (e.g., avoiding defeat).

There is also interest in this area of study in the business world, where it is common to find interventions intended to “increase employee motivation”. From this perspective, motivation is seen as a dynamic process that can vary over time. This view is not incompatible with the many studies that advocate concentrating on ways of tackling any challenge motivationally, particularly in the world of work. Thus, just as we commonly speak of the existence of certain individual personality traits, we might also identify different motivational traits.

One example is the theory developed by Kanfer and Heggestad [11], who differentiated between proximal influences on performance—such as individual differences in self-regulation or skills—and distal influences—reflecting certain stable motivational traits. Specifically, the authors identify three principal motivational traits: personal mastery, competitive excellence, and motivation related to anxiety, each which is in turn made up of three underlying factors.

The authors have created several batteries of items to measure these motivational traits. A short form of their Motivational Trait Questionnaire (MTQ-S) [12] distils the underlying factors from nine to six, with two associated with each major trait factor.

Thus, personal mastery is made up of desire-to-learn and mastery. The difference between the two components is that whereas the former refers to the acquisition of new skills and knowledge, the latter involves improving existing skills. Both may therefore be considered to be approach-oriented.

The second factor, competitive excellence, comprises a mixture of goal orientations. It involves an element of comparison with others, since other-referenced goals use other people’s performance as a benchmark for assessing the individual’s own performance, while the competitiveness component adds the desire to surpass others. Both components contain approach aspects [13], but the former can also be seen as a form of avoidance orientation (i.e., comparing oneself to others in order not to do things too badly and be punished).

Finally, motivation related to anxiety is entirely avoidance-oriented. Worry refers to the apprehension a person feels in the workplace, while emotionality encompasses the wider emotions that may appear in a context of performance evaluation.

This last concept (performance) has also been extensively researched in recent decades, given its relationship with aspects such as company survival and job maintenance.

In 2011, a team led by Koopmans [14] drew on medical, psychology, and business management databases to carry out a systematic review of the literature on individual work performance. The result was a conceptual framework that identified four separate dimensions: task performance, contextual performance, adaptive performance, and counterproductive performance. This approach enables more aspects to be considered than simple productivity and offers a theoretical explanation for the value certain people may have within a company, despite not being the best performers in specific tasks.

Like motivation, two of these dimensions can be seen in terms of approach or avoidance, taking the organization itself as a reference point. Firstly, contextual performance entails individual behaviors that support the organizational, social, and psychological environment in which the tasks are performed; in other words, it does not refer to job-related aspects. Other ways of viewing this type of performance include organizational citizenship behaviors (OCBs), organizational prosocial behaviors, or extra-role behaviors.

The opposite aspect—although Coyne et al. [15] caution against referring to two bipolar dimensions—is counterproductive performance or counterproductive work behaviors (CWBs). Although this factor has not been as extensively studied, it is easy to recognize in any business environment, in aspects such as absenteeism and presenteeism. It therefore constitutes deliberate behavior that is harmful for the organization.

The relationships between motivation and performance have been extendedly studied. Most research concludes that motivation predicts several outcomes, including performance. The intuitive result (more motivation lead to better performance) has been found in both educational [16] and organizational context [17], through different contexts and even species [18]).

But some previous studies have concluded that the relationships may be more complicated. Thereby, previous research has concluded that autonomous motivation (like approach orientation) is positively related to performance, meanwhile controlled motivation (like avoidance orientation) and amotivation are negatively related to performance [19]. Another research has revealed that avoidance motivation is positively related to workplace deviance, meanwhile the relationships between approach motivation and deviance is even more complex [20].

In view of all the foregoing, this study seeks to examine in greater depth the possible relationship between motivational traits and performance, defined in this paper in terms of organizational citizenship behaviors (positive performance) and counterproductive behaviors (negative performance). Specifically, we propose the following hypotheses:

**H1.**  *Motivational traits are directly related to performance as follows:*

**H1a.**  *Motivational traits of “approach” are positively related to organizational citizenship behaviors.*

**H1b.**  *Motivational traits of “avoidance” are positively related to counterproductive behaviors.*

In addition, we expect that the sizes of the effects will be larger for H1a than H1b.

### 1.2. The Mediating Role of Psychological Capital Resources

Among the different studies of extra-role behavior, Luthans [21] (p. 59) defines positive organizational behavior, that we can consider OCBs synonymous, as “the study and application of positively-oriented human resource strengths and psychological capacities that can be measured, developed, and effectively managed for performance improvement in today’s workplace”. The same author [22] uses confidence, hope, and resilience as criteria for measuring this type of behavior.

Together with optimism, these constructs constitute the higher-order factor of psychological capital, or PsyCap, which is defined [23] (p. 3) as being “an individual’s positive psychological state of development that is characterized by: (1) having confidence (self-efficacy) to take on and put in the necessary effort to succeed at challenging tasks; (2) making a positive attribution (optimism) about succeeding now and in the future; (3) persevering toward goals and, when necessary, redirecting paths to goals (hope) in order to succeed; and (4) when beset by problems and adversity, sustaining and bouncing back and even beyond (resilience) to attain success”.

The hierarchical factor structure for PsyCap is broadly accepted and it seems to be valid cross-culturally [24], and even though Wernsing [24] propose a simplified conceptual version of PsyCap (hope, self-efficacy, and resilience), the four first-factor latent constructs are frequently used in investigations. Although the factors have been also studied separately, especially self-efficacy, PsyCap has gotten attention from investigators, probably due to its broader definition that involves relationships with immediate in-role productivity, but also with several desired and undesired behaviors, attitudes, and intentions [25].

In recent years, numerous authors have taken an interest in this concept, testing its relationship with different variables such as satisfaction (i.e., Jafari and Hesampour [26], Luthans et al. [27], or Etikariena [28]), performance (i.e., Rabenu et al. [29], Carmona-Halty et al. [30], or Madrid [31]), and motivation (i.e., Kim and Noh [32]; Ferraro et al. [33]; Datu et al. [34]; or Siu et al. [35]) in different contexts and social groups. Some of these studies, such as the ones by Etikariena [28] and Carmona-Halty et al. [30], posit a mediating role for psychological capital—in the case of the former, with regard to the relationship between satisfaction and innovating behavior at work; and in the latter, with regard to the relationship between positive emotions and academic performance. Both studies offer evidence to support this hypothesis.

Based on these studies, we posit the hypothesis that psychological capital is related to both motivation and performance. Specifically, we propose that:

**H2.**  *The relationship between workers’ motivational traits and performance will be mediated by the individual’s psychological capital resources.*

However, according to Luthans et al.’s results [36], this mediation probably does not function in the same way in all possible relationships, being more important in and more closely related to behaviors that pursue a benefit for the organization. We thus propose that:

**H3.**  *The mediation of psychological capital resources will be especially relevant for predicting positive performance (organizational citizenship behaviors).*

According to the first hypothesis, we expect that the sizes of the effects of motivational traits on psychological capital resources, will be larger for the approach orientation traits.

**Figure 1:**
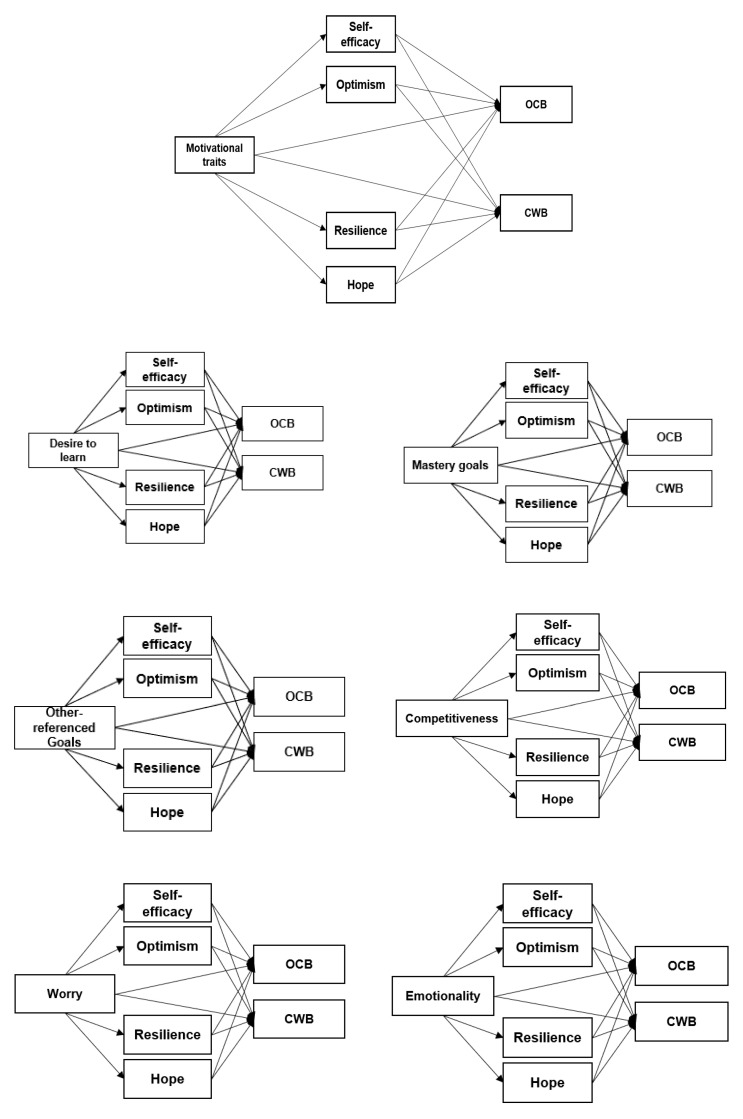
General mediation model proposed and tested models.

The general hypothesis of this study is shown in the Figure 1 (the third hypothesis, as well as hypothesis 1.a and 1.b, are better analyzed in the figures in the Results section).

## 2. Materials and Methods

### 2.1. Ethical Information

The Bio-Ethics Committee of the National Distance Education University approved the study protocol in accordance with the Declaration of Helsinki (protocol number 160504). In the present study, potential participants were informed of the aims of the research being carried out and the conditions regarding anonymity and the voluntary nature of their collaboration. They were also assured they could withdraw from the study at any time without penalty. The only criteria for participating were being older than 40 years of age and having employee status. The main reasons to include these criteria were: a) the aging trends of workplace make middle adulthood an interesting stage of human development to analyze, and b) job instability or not being part of the organization could distort motivational results. Those who finally decided to participate signed a consent form and completed booklets containing the research questions.

### 2.2. Participants

The study sample was made up of employees aged over 40 years who were resident in Spain. A total of 741 volunteers (50.2% female) answered our questionnaire. The average age was 49.04 (SD, standard deviation = 6.43) years, and the average length of time working in the company was 16.33 (SD = 10.98) years. In terms of professional category, participants included regular employees (61.6%), middle managers (17.2%), and executives or owners (17.6%) (the remaining 3.6% failed to public-sector companies (29.9%), with the remainder working in private foundations or companies linked to the public sector. In terms of education level, 40.7% had a university degree, 18.2% had vocational training qualifications, and 17.3% has completed the Spanish baccalaureate (the remaining 1.9% did not answer this question).

### 2.3. Procedure

In order to achieve the aims of the study, a homogeneous purposive sampling was used, focusing on those workers over 40. Data were collected at two different points in time (with a 4-month interval between the two), because the response rate was low (17.44%). We contacted potential participating companies through HR consultancy firms (public and private) associated with the university through research agreements. The organizations were informed clearly and accurately about the purpose of the research, the procedure to be used, and the time required to perform the questionnaire. Companies agreeing to collaborate were sent the questionnaires (response rate 38.46%). They then distributed them among employees aged over 40 (at both moments in the data gathering process), who completed them on a voluntary basis; all their workers who met the criteria were offered the opportunity to participate in the study. Participants completed the registration page and the consent form first and filled out the questionnaire second. To ensure confidentiality, participants created a personal code especially for the study and submitted the completed questionnaire to the collaborating organizations in a sealed envelope.

### 2.4. Instruments

#### 2.4.1. Motivational Traits

We used a translated version of the Motivational Traits Questionnaire Short Form (MTQ-S) by Kanfer and Ackerman [12]. The questionnaire has a 5-point Likert-type response scale and is divided into 6 sub-scales, one for each motivational trait. Examples of items include: “When I am learning something new, I try to understand it completely” (desire to learn–approach); “I like to turn things into a competition” (competitiveness–approach); and “I worry about the possibility of failure or underperforming” (worry–avoidance). The sub-scales form paired dimensions, with the following reliability levels: personal mastery 0.802, competitive excellence 0.876, and motivation related to anxiety 0.864.

#### 2.4.2. Psychological Capital

We used a Spanish adaptation of the Psy Cap Questionnaire [23], in which participants are asked to rate, on a 5-point Likert-type scale, their level of agreement with items related to self-efficacy (α = 0.83) (“I feel confident helping to set targets/goals in my company”) (a), hope (α = 0.61) (“In general, I think there are lots of ways around any problem”) (b), resilience (α = 0.70) (“I feel I can handle many things at a time at this job”) (c), and optimism (α = 0.60) (“I always look on the bright side of things regarding my job”) (d). The reliability of the instrument in this study was 0.828. Some of the subscales did not have high internal consistency in our sample; however, Schmitt [37] has argued that when a scale has other desirable properties, such as meaningful content coverage, a relatively low alpha coefficient is not necessarily an impediment to its use.

#### 2.4.3. Performance

Our interest in this variable centers on behaviors that are not directly related to the tasks of the job, but which have an impact on the organization. We therefore used a translated and adapted version of the Counterproductive Work Behavior Checklist (CWB-C) developed by Spector et al. [38], which defines a series of both positive (α = 0.64) (e.g., “Invest extra effort and keep going until tasks are successfully completed”) and negative behaviors (α = 0.82) (“Pretend to be sick to avoid carrying out certain tasks”). This version comprises a total of 10 items (4 related to organizational citizenship behaviors and 6 to counterproductive behaviors), measured on a 5-point Likert-type scale.

### 2.5. Statistical Analyses

To test our hypotheses, we used the PROCESS macro for SPSS, it may be downloaded from the website processmacro.org [39]. We applied mediation model 4, which estimates the relationship of X (motivational traits) to Y (performance) with the mediation of M (psychological capital) in the relationship X→Y (motivational traits → performance). The procedure was based on 5000 bootstrap samples, with a confidence interval of 95%. The procedure enables us to estimate: (1) the relationship of the independent variable to the different mediators through different models; (2) the overall relationship between the independent variable and the dependent variable, including the mediating variables and the covariables; and (3) the direct relationship between the independent variable and the variable, and the indirect relationship through the possible mediating variables. In order for the mediation hypotheses to be accepted, the indirect relationships between the independent and dependent variables and the relationship between these variables and the mediating variables must be statistically significant. When the zero is not included in the confidence interval at 95%, it can be concluded that the parameters are significantly different from zero at *p* < 0.05. The covariables used in the analysis were age and length of time working in the company.

## 3. Results

Before testing our models, we performed a correlation analysis among the variables studied, as well as indicators’ Collinearity Statistics. All Variance Inflation Factor (VIF) values are lower than 5 and their tolerance values are higher than 0.2, so there is no collinearity problem. The results are shown in Table 1.

On the one hand, the Pearson correlations revealed that all significant relationships were in the predicted directions. As shown, the highest correlations are revealed between the motivational traits underlying the main traits (personal mastery, competitive excellence, and motivation related to anxiety). Some of these correlations supported stereotypes regarding older workers, while others indicated strengths among this group, such as less worry and fewer counterproductive behaviors, possibly due to their greater experience. Consistently with our hypotheses, all PsyCap dimensions are positively related to organizational citizenship behaviors and negatively related to counterproductive behaviors. The relationships between motivational traits, PsyCap, and performance vary in direction from case to case.

On the other hand, some of the variables showed low reliability. Although values higher than 0.70 are recommended, some authors states that, for exploratory studies, values between 0.60 and 0.70 are considered acceptable [40]. Keeping this information and the instruments’ aggregated reliability levels in mind, and even though the values of the scales of hope, optimism, and CWB-C should be improved, we went ahead with the investigation.

### Mediation Analysis

The goal of this analysis was to test our working hypotheses. We therefore built a series of models to relate each of the scales in the MTQ to organizational citizenship behaviors and counterproductive behaviors. In all models, we included age and length of time working in the company as control variables. To aid interpretation, the figures show only the significant relationships, and the mediation relationships are shown in thicker lines.

**Figure 2:**
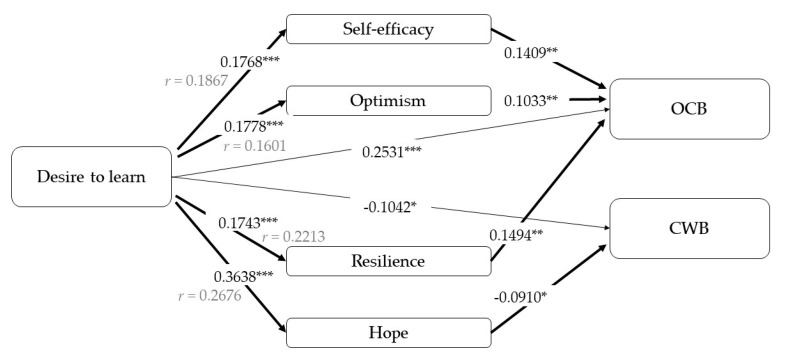
Results of the mediation models with desire to learn as an independent variable. Note: [95% CI]; * *p* < 0.05; ** *p* < 0.01; *** *p* < 0.001.

The first model (Figure 2) shows a positive relationship between desire to learn and OCBs and a negative relationship with CWBs, which support our first analysis. Moreover, desire to learn is related to all the PsyCap dimensions. Regarding our mediational hypothesis on OCBs, the total effect is statistically significant (B, beta coefficient = 0.31, *p* < 0.001, *r* = 0.29), as well the direct effect and the indirect effect of desire to learn through efficacy (B = 0.02, 95% CI, confidence interval [0.00; 0.06]), optimism (B = 0.01, 95% CI [0.00; 0.05]), and resilience (B = 0.03, 95% CI [0.01; 0.06]).

In contrast, in the relationship with CWBs, hope is the only factor that is significantly related, in this case negatively (B= −0.09, 95% CI [−0.17; −0.01]). Again, the mediational hypothesis is confirmed in this case (total effect: B = 0.14, *p* < 0.001, *r* = 0.17; indirect effect though hope: B= −0.03, 95% CI [−0.07; −0.01]).

**Figure 3:**
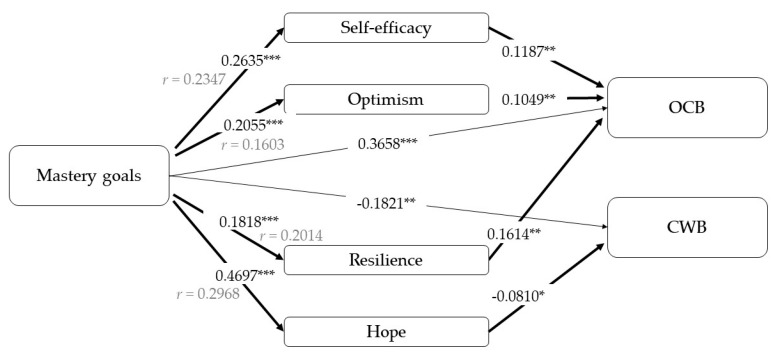
Results of the mediation models with mastery goals as an independent variable. Note: [95% CI]; * *p* < 0.05; ** *p* < 0.01; *** *p* < 0.001.

Secondly, the other underlying factor of personal mastery, mastery goals (Figure 3), shows similar relationships to desire to learn. On the one hand, the direct effect of mastery goals on OCBs and CWBs is confirmed, showing a positive relationship with OCBs and a negative one with CWBs. On the other hand, it is revealed a positive relationship between the mastery goals and all PsyCap dimensions.

Regarding to mediational analyses, total effect is statistically significant in both cases, OCBs model (B = 0.43, *p* < 0.001, *r* = 0.40) and CWBs model (B = −0.23, *p* < 0.001, *r* = 0.20). Repeating the previous results, the indirect effect of mastery goals on OCBs would being explained through efficacy (B = 0.03, 95% CI [0.01; 0.07]), optimism (B = 0.02, 95% CI [0.00; 0.06]) and resilience (B = 0.03, 95% CI [0.01; 0.06]). Additionally, hope appears as a mediator in the relationship between mastery goals and CWBs (B = −0.04, 95% CI [−0.07; −0.01]).

**Figure 4:**
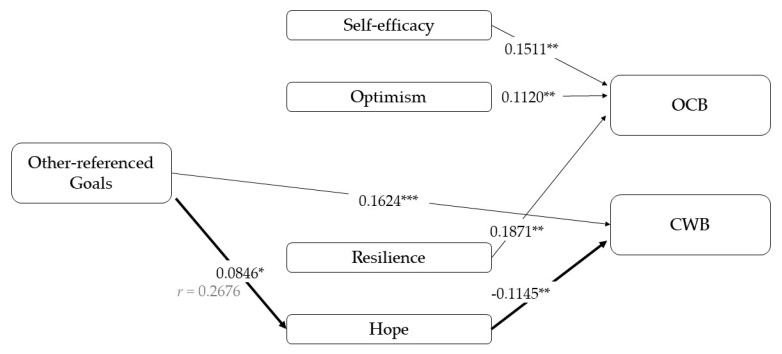
Results of the mediation models with other-referenced goals as an independent variable. Note: [95% CI]; * *p* < 0.05; ** *p* < 0.01; *** *p* < 0.001.

Thirdly, other-referenced goals dimension (Figure 4) has no statistically significant relationship with OCBs, but the direct effect on CWBs is confirmed. In respect of the relationships with PsyCap, it just shows a significant relationship with other-referenced goals and a marginal relationship with efficacy (B = 0.06, *p* = 0.06). The mediational analysis regarding to CWBs is confirmed through hope (B = −0.01, 95% CI [−0.02; −0.00]; total effect: B = 0.15, *p* < 0.001, *r* = 0.19).

**Figure 5:**
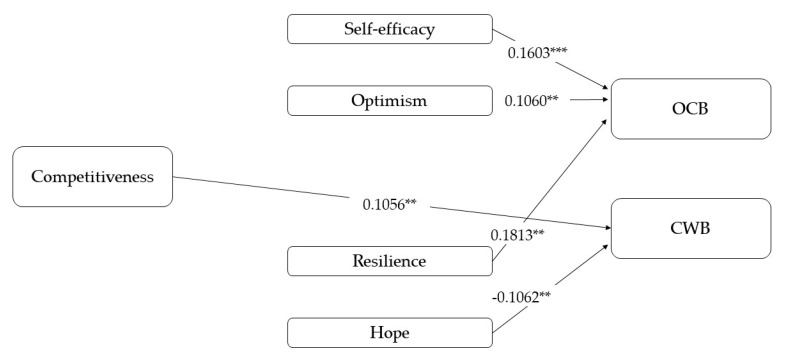
Results of the mediation models with competitiveness as an independent variable. Note: [95% CI]; ** *p* < 0.01; *** *p* < 0.001.

The fourth model analyses the relationships between competitiveness and the other variables (Figure 5). As in the previous model, the results reveal no direct relationship between competitiveness and OCBs, but the direct effect on CWBs is confirmed by the data (total effect *r* = 0.15). In this case, competitiveness does not appear to be a good predictor of none of the PsyCap variables (only self-efficacy shows a marginal relationship with competitiveness, *p* = 0.08), which determine that mediation hypothesis is not supported.

**Figure 6:**
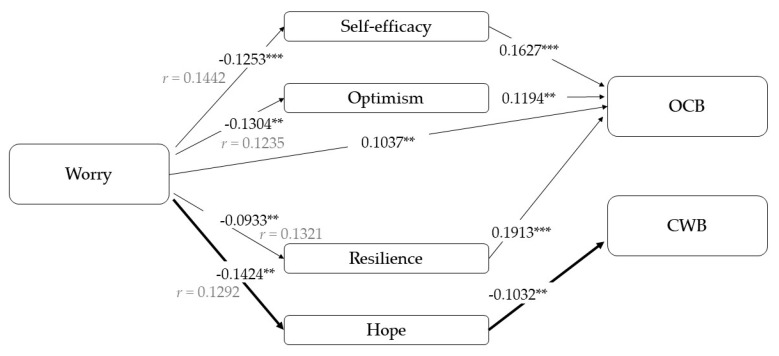
Results of the mediation models with worry as an independent variable. Note: [95% CI]; ** *p* < 0.01; *** *p* < 0.001.

Next, we analyzed the relationship between worry and performance (Figure 6). Worry is associated, albeit negatively, with all PsyCap components. Regarding mediational analyses, and although there is no direct effect of worry on CWBs (*p* = 0.15), we found an indirect effect through hope (B = 0.01, 95% CI [0.00; 0.04]; marginal total effect: B = 0.08, *p* = 0.06). The more surprising result is related to OCBs: while direct effect of worry on those behaviors is positive and statistically significant, and the total indirect effect through efficacy, optimism, and resilience is confirmed (B = −0.06, 95% CI [−0.09; −0.02]), the total effect of worry on OCBs is not statistically relevant (B = 0.05, *p* = 0.24).

**Figure 7:**
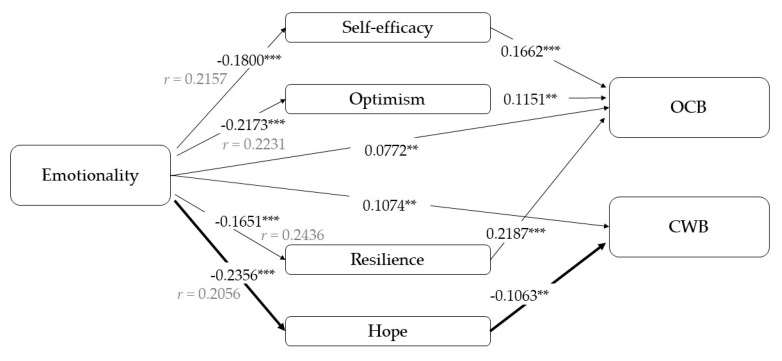
Results of the mediation models with emotionality as an independent variable. Note: [95% CI]; ** *p* < 0.01; *** *p* < 0.001.

Finally, we analyzed the models that take emotionality as an independent variable (Figure 7). This factor was found to be significantly and negatively associated with all PsyCap components. As with the other component of motivation related to anxiety, we confirm the existence of a direct effect of emotionality on OCBs (B = 0.08, *p* = 0.04) and indirect effect through efficacy, resilience, and optimism (total indirect effect: B = −0.09, 95% CI [−0.13; −0.06]), but the total effect on OCBs is not statistically significant (B = −0.01, *p* = 0.70).

Otherwise, both the total (B = 0.13, *p* < 0.001, *r* = 0.18) and direct effect of emotionality on CWBs are confirmed, as well as the mediational pattern through hope (B = 0.03, 95% CI [0.01; 0.05]).

The control variables behaved similarly in all models, with a significant negative relationship being observed between length of time working in the company and organizational citizenship behaviors, and a significant negative relationship being observed between age and counterproductive behaviors.

In sum, the results of hypotheses testing are shown in Table 2.

## 4. Discussion

Our first conclusion is that the relationships between different motivational traits and PsyCap dimensions and organizational citizenship behaviors are not the same, i.e., not all personal traits and resources have the same influence on the appearance of behaviors that benefit or harm the company. In order to make the results clear, we discuss the results according to the main motivational traits factors.

### 4.1. Principal Findings

In order to present this section clearly, the principal findings are organized by motivational traits orientations, and subsequently, a recapitulation is made.

#### 4.1.1. Approach Traits

To begin with the motivational traits that appear to be most closely related to the performance dimensions, both OCBs and CWBs, it is noteworthy that both desires to learn and mastery goals are positively associated with all PsyCap dimensions. Both of these motivational traits reflect a “long-term” vision, involving individuals in tasks whose achievement will be related to their keenness to begin, but also to their capacity to continue contributing resources to achieve their goal.

Moreover, both traits are directly related to performance—positively in the case of organizational citizenship behaviors and negatively in the case of counterproductive behaviors, showing up larger sizes of effects on OCBs than CWB. The effect is certainly large in the case of mastery goals and OCBs [41]; it could mean that the pursuit of perfectionism is somehow big-hearted, improving not just abilities or skills but the environment. These relationships seem logical since both desires to learn and mastery goals are approach traits, which are associated with performance improvement understood in the broadest sense of the term but being more relevant doing good than simply avoiding bad behavior.

The mediation of PsyCap dimensions again indicates an association between these concepts and positive results, since three of them (self-efficacy, resilience, and optimism) lie behind the relationships between the two scales of personal mastery and organizational citizenship behaviors. One analysis—which concurs with the existing literature [42]—might be that having greater personal resources can make it easier to obtain better organizational results. Those findings appear consistently on different cultures and workplaces [43,44,45], and PsyCap resources are relevant for performance as well when they are analyzed separately [46]. Another analysis might be that approach motivational traits may be based on psychological resources. This could mean that an improvement introduced through programs to promote psychological capital could affect motivations and, ultimately, performance [47].

As for the relationship between the two scales and counterproductive behaviors, hope is the only factor that mediates in the relationship, acting as a “protection” factor, since counterproductive behaviors will more readily appear when individual hope is low. This result is consistent with previous studies where hope differs from the other dimensions of PsyCap when relating to other variables [48]. It would seem plausible that when an optimistic motivational state exists with regard to goals and the possibilities of achieving them, behaviors that are harmful to the company are avoided, whereas in its absence, individuals are much less engaged with the organization.

Regarding the last approach motivational trait, competitiveness, the relationship with PsyCap is inexistent. The absence of relationship between competitiveness and all the PsyCap dimensions could mean that the pursuit of beating others is not about the personal resources, but a psychological need. This individual-centered perspective fits with the also absent relationship with OCBs: a selfish behavior is not interested in fostering a healthier organization. Moreover, our results support previous studies where frustration of basic psychological needs like competence was related to CWBs [49].

The significance of this relationship is that the aim for people who are competitiveness oriented, is winning, even if it involves pit himself against other [50]. This result is also consistent with the absence of relationship with PsyCap resources: if the objective is winning it can be reached by cheating, excellence is not needed.

#### 4.1.2. Mixed Trait

The only one trait that we addressed as mixed is other-referenced goals, which, unlike competitiveness, is positively related to psychological capital resources, but only with hope. In line with our above ideas, this result could be explained due to the difference between other-referenced goals and competitiveness. One explanation could be that, although both terms imply comparison with other, other-referenced links with the hope of at least, reach the accomplishment desired, while competitiveness not. It is easier to define a fair performance than a superior performance and it could explain that other-referenced goals is linked to hope: to be able to clearly visualize the objective is useful to redirect efforts towards the desired direction.

Like competitiveness, this trait is not an effective predictor of organizational citizenship behaviors; however, like competitiveness, is related to counterproductive behaviors. In this case, its relationship with hope consists of mediating the overall relationship with such behaviors. This mediation is again negative; thus, in this case too, hope acts as a protector against the onset of counterproductive behaviors towards the organization. When people compare themselves to others, having a positive attitude can keep them attentive to their work, whereas in a situation of comparative hopelessness and insecurity, negative behaviors might appear towards the organization in general terms and towards the work team in particular.

In sum up, taking both competitive excellence traits, it therefore appears that the simple act of comparing can have negative consequences for the organization, even without requiring competition. Those results contrasts with previous studies where competitiveness was related to a better performance in different tasks [51,52]; the main difference is that those studies normally explore the relationship with task performance, not with extra-role performance. This is important when it comes to managing teams, where individual comparisons are perhaps less useful, and using inter-group approaches might serve to derive benefits by sharing common goals. In this case, we cannot uphold the hypothesis of mediation by psychological capital, and therefore other variables may be involved in the direct relationship between this motivational trait and counterproductive behaviors. The relationship could be better explained by contextual variables, such as the value attached by the company to direct competition, other personality variables such as narcissism or a results-oriented approach or feelings of envy [53].

#### 4.1.3. Avoidance Traits

One very revealing result is that of worry, which is negatively associated with all dimensions of PsyCap and is positively related to OCBs, but not with CWBs. The result analyzing the mediational hypothesis, it is the fact that the effect on OCBs is cancelled by the effect through PsyCap, which endorse with previous founds about worrying [54], while the total effect on CWBs is produced through hope. We can therefore conclude that worry acts as a “disabling” motivator in order to improve the performance, since it does not foster the appearance of organizational citizenship behaviors. Nevertheless, it is related to the appearance of CWBs, which seems paradoxical since worry concerns “doing things badly”, and it is assumed that acting negatively towards the company would be inconsistent with that. Those results could mean that worry can affect decision making [55] or even is related to certain Machiavellianism, seeking for the personal benefit even though it can be harmful for the organization [56]. An alternative explanation is that the protective power of hope is not enough to counter the negative impact of worry.

The other component of motivation related to anxiety, emotionality, is also negatively associated with all components of psychological capital, and we can therefore conclude that individuals who score highly in avoidance motivational traits may hide a lack of personal resources when it comes to coping with tasks.

Again, this avoidance factor is relevant for explaining the appearance of counterproductive behaviors [20]. The difference with worry is that whereas worry is concerned with doing something badly, emotionality refers directly to the negative emotions experienced by the worker. The relationship of this term with neuroticism should be noted, and this personality trait has been reported as a CWB predictor [57]. This aspect is much more harmful for the individual, who may come to view the organization as being to blame for those consequences and take reprisals in the form of counterproductive behaviors. Once again, hope acts as a mediator. One possible explanation for this finding is that the invigorating attitude entailed by hope, together with a high degree of emotionality, which has negative consequences, would lead the individual in question to act erroneously (e.g., taking company material away “to work at home”), even when pursuing the benefit for the company [58]. It is also possible that hope gives rise to a certain sense of invulnerability (e.g., “given that I am reaching my targets, it is not important to be on time.”) which, combined with a high degree of emotionality, may result in the rationalization of harmful behavior towards the company (e.g., “the important thing is for me to get a good night’s rest and arrive with my batteries charged.”).

Those results present motivation related to anxiety as not desirable traits in our workers. However, other studies find the good side of anxiety, giving it a role as an effective motivator [59,60]. The interactions are certainly complex [61], with emotional intelligence as a potential moderator [62,63].

#### 4.1.4. Recapitulation

Thus, our results provide at least partial empirical evidence to support the working hypotheses posited. Specifically, they reveal that motivational traits are related directly and indirectly to performance through psychological capital, although such relationships depend on motivational traits and specific behaviors.

The approach-oriented motivational traits are found to have a positive relationship with PsyCap and are therefore related more to positive than to negative performance. The role of PsyCap in relation to counterproductive behaviors is mainly “protective” in nature.

As for the control variables, the results reveal that the older a person is, the less likely they are to display counterproductive behaviors; this could be proof of the idea that “experience is worth as much as a degree” and that such individuals avoid creating situations in which their performance might be viewed negatively, with the threat of dismissal being even greater for older people who may have more difficulty finding another job. The length of time a person has been employed in the organization also appears to be relevant; it is possible that while newcomers are eager to “please” their colleagues, this motivation declines over time or people just get tired of worrying about creating a pleasing workplace [64].

### 4.2. Directions for Further Research

Those results can be followed up by studies, which shed more light on the existing relationships between motivational traits, PsyCap, and performance. In this research, some motivational traits appear more deeply related to PsyCap and performance, i.e., personal mastery. Researching about if it is possible to promote this kind of motivation even among the most avoidance-oriented workers could be an interesting field, with useful practical consequences, not only for organizations even for the workers’ health and wellness.

One aspect to be discussed is the possibility that some contextual variables may affect those relationships, e.g., the expansion of gig economy or teleworking, where characteristics like age [65], gender [66], or design of the labor platform [67] can be crucial to define the motivation and orientation towards work performance.

In addition, it would be recommended to explore even more group characteristics that influence those relationships such as resources as a team [68], team identity [69], or the leadership impact [70].

### 4.3. Implications for Practice

One of the main contributions made by this study is that it establishes relationships between a series of approach and avoidance motivational traits and certain results for the organization as a whole. These relationships are mediated by PsyCap resources, a finding which suggests that it may be possible to improve performance through these resources.

One way of developing employee motivation at a cognitive level would be to understand situations more positively, reinforcing ideas of approach over those of avoidance. In the medium-term, this might create “self-fulfilling prophecy”-type situations mediated by PsyCap (e.g., “I am going to achieve the best results in the office because I am capable of it.”). This objective could be attained through emotional intelligence development, preventing from emotional exhaustion [71], which reinforce the habitual formations in order to improve social skills. This kind of formation can be useful because of the potential impact on self-efficacy and emotion management [72], which according with our results would increase OCBs and prevent CWBs.

Moreover, it is likely that motivational traits, despite being long-term trends, may also be modified in some way by the development of PsyCap resources, leading to a potential spiral of improvement not only in performance, but in the worker’s own satisfaction too.

A crucial managerial implication is addressed on this paper: not every worker is equal. This statement could seem empty, but it is still necessary to be marked. The differences are not only referred to capabilities but to attitudes and some of them respond to personal traits, and they affect the possible performance [73]. Understanding how workers in charge process the information and set goals is important to help the team to attain them. Communication becomes a key factor to motivate workers, and previous research has shown the differential effectiveness of performance feedback considering personality traits and task demands [74]; adding these results to ours (motivational orientation and contextual demands), individualized consideration from the leader may be mandatory.

This implication is related with another aspect mentioned in this paper: the negative consequences that competence can origin within organizations. Unhealthy competitiveness may have long term negative consequences; i.e., being rejected by other group members [75]. According to the findings of this paper and taking into account that comparison is probably inevitable among workers, it is recommended to set the objectives in advance and based on personal characteristics.

### 4.4. Limitations of the Study

This study has several limitations that should be acknowledged. Firstly, it should be noted that the measurement of performance used in this study does not refer to task performance. It is possible that the relationship between PsyCap resources—and particularly motivational traits—and performance may vary depending on the measure used. Even more, some variables present improvable levels of reliability, maybe other instruments should be used in future researches.

Moreover, the sample used may be affected by the measurements themselves. For example, we surveyed different types of employee. Questions about competitiveness may be “misunderstood” and it may be perceived negatively by some employees, while others—such as those in an executive role—may see it as something positive and even necessary.

Another limitation involves the selection process, which was not random but rather based on convenience sampling. This is together with the fact that our participants aged over 40 may have skewed the results and likely do not reflect the impact in the general population.

It may not be possible to extrapolate our findings to other cultures or organizational contexts, due to their possible differences. One example is that of an employee who does overtime to complete a job; in some cultures, this individual may be seen as being highly motivated, whereas in others this practice may be viewed as reflecting an inefficient use of regular working hours.

Finally, we use self-report measures, which always involve the risk of the social desirability bias.

## 5. Conclusions

This paper provides evidence of the advantage for organizations of having employees with specific individual motivational traits that are related to positive organizational consequences. These relationships are mediated by PsyCap, and therefore an intervention geared towards developing individual aspects such as self-efficacy and resilience may impact the organizational results both directly and indirectly (through work motivations).

## Figures and Tables

**Table 1:** Descriptives, Pearson Correlations, and Indicators’ Collinearity Statistics.

Variables	M	SD	VIF	1	2	3	4	5	6	7	8	9	10	11	12	13	14	15
Age	49.03	6.73	n/a	n/a														
Gender	1.50	0.50	n/a	−0.002	n/a													
Years in company	16.33	10.98	n/a	−0.492 **	−0.072	n/a												
Desire to learn	3.96	0.55	1.889	−0.073 *	−0.015	−0.023	0.651											
Mastery goals	3.60	0.47	2.004	−0.103 **	−0.003	−0.089 *	0.663 **	0.700										
Other-referenced goals	2.86	0.69	2.271	−0.128 **	−0.047	−0.116 **	0.097 **	0.214 **	0.845									
Competitiveness	2.5	0.62	1.940	−0.138 **	−0.149 **	−0.114 **	0.009	0.181 **	0.651 **	0.755								
Worry	3.26	0.57	2.063	−0.102 **	0.178 **	−0.104 **	0.084 *	0.064	0.365 **	0.083 *	0.757							
Emotionality	2.87	0.63	2.872	−0.031	0.181 **	−0.047	−0.065	−0.072	0.303 **	0.066	0.672 **	0.801						
Self-efficacy	3.60	0.55	1.333	0.010	−0.071	−0.051	0.172 **	0.225 **	0.075 *	0.070	−0.124 **	−0.202 **	0.651					
Hope	3.72	0.77	1.510	−0.053	0.012	−0.078 *	0.259 **	0.294 **	0.084 *	0.047	−0.092 *	−0.187 **	0.401 **	0.610				
Resilience	3.76	0.45	1.381	0.035	0.029	−0.023	0.210 **	0.191 **	0.032	−0.037	−0.116 **	−0.231 **	0.367 **	0.414 **	0.678			
Optimism	3.60	0.62	1.261	−0.020	0.051	−0.034	0.158 **	0.159 **	−0.050	−0.019	−0.113 **	−0.219 **	0.273 **	0.393 **	0.293 **	0.496		
Positive performance	3.76	0.64	1.354	−0.028	−0.012	−0.112 **	0.268 **	0.327 **	0.024	−0.061	0.056	−0.006	0.222 **	0.170 **	0.215 **	0.189 **	0.537	
Negative performance	1.66	0.68	1.100	−0.122 **	0.004	−0.020	−0.104 **	−0.142 **	0.0161 **	0.100 **	0.081 *	0.126 **	−0.077 *	−0.132 **	−0.069	−0.086 *	−0.078 *	0.822

Note: Gender (1 = Male); Values in the diagonal are reliabilities of the variables. * *p* < 0.05; ** *p* < 0.01; n/a: not applicable.

**Table 2:** Results of hypotheses testing (*n* = 741).

**Model with Direct Effects**						
Desire to learn	OCB	*H1*	Supported	*H1a*	Supported	
	CWB		Supported			
Mastery	OCB	*H1*	Supported	*H1a*	Supported	
	CWB		Supported			
Other Referenced Goals	OCB	*H1*	Not supported	*H1a*	Not supported	
	CWB		Supported	*H1b*	Supported	
Competitiveness	OCB	*H1*	Not supported	*H1a*	Not supported	
	CWB		Supported			
Worry	OCB	*H1*	Supported	*H1b*	Not supported	
	CWB		Not supported			
Emotionality	OCB	*H1*	Supported	*H1b*	Supported	
	CWB		Supported			
**Model with Indirect Effects**						
Desire to learn	Self-efficacy	OCB	*H2*	Supported	*H3*	Supported
	Optimism			Supported		
	Resilience			Supported		
	Hope	CWB		Supported		
Mastery	Self-efficacy	OCB	*H2*	Supported	*H3*	Supported
	Optimism			Supported		
	Resilience			Supported		
	Hope	CWB		Supported		
Other Referenced Goals	Self-efficacy	OCB	*H2*	Not supported	*H3*	Not supported
	Optimism			Not supported		
	Resilience			Not supported		
	Hope	CWB		Supported		
Competitiveness	Self-efficacy	OCB	*H2*	Not supported	*H3*	Not supported
	Optimism			Not supported		
	Resilience			Not supported		
	Hope	CWB		Not supported		
Worry	Self-efficacy	OCB	*H2*	Not supported	*H3*	Not supported
	Optimism			Not supported		
	Resilience			Not supported		
	Hope	CWB		Supported		
Emotionality	Self-efficacy	OCB	*H2*	Not supported	*H3*	Not supported
	Optimism			Not supported		
	Resilience			Not supported		
	Hope	CWB		Supported		
